# *In Vitro* Assessment of Cytokine Expression Profile of MCF-7 Cells in Response to hWJ-MSCs Secretome

**DOI:** 10.15171/apb.2019.075

**Published:** 2019-10-24

**Authors:** Mansoureh Mirabdollahi, Shaghayegh Haghjooy Javanmard, Hojjat Sadeghi-Aliabadi

**Affiliations:** ^1^Applied Physiology Research Center, Cardiovascular Research Institute, Department of Physiology, School of Medicine, Isfahan University of Medical Sciences, Isfahan, Iran.; ^2^Medicinal Chemistry Department, School of Pharmacy, Isfahan University of Medical Sciences, Isfahan, Iran.

**Keywords:** Human Wharton’s Jelly mesenchymal stem cells (hWJ-, MCF-7, Tumor cells, Secretome, Cytokine expression profile

## Abstract

***Purpose:*** Several attempts have been made to identify the mechanisms by which mesenchymal stem cells (MSCs)-derived secretome exert anti-tumor or tumorigenic effects, but still further investigations are needed to explore this subject. Thus, in this study we want to examine the expression of different cytokines in secretome of hWJ-MSCs and their effects on cytokine expression profile of the MCF-7 tumor cells.

***Methods:*** The hWJ-MSCs were isolated and characterized according to the International Society for Cellular Therapy criteria. Then, secretome of hWJ-MSCs was collected and freeze-dried, and 20 mg/mL of the freeze-dried secretome was used to treat MCF-7 cancer cells for 48 hours. Afterwards, the expression levels of 12 cytokines including IL-1a, IL-1b, IL-2, IL-4, IL-6, IL-8, IL-10, IL-12, IL-17A, TNFα, IFNγ and GM-CSF in secretome of hWJ-MSCs alone as well as in supernatant of tumor cells before and after treatment with hWJ-MSCs secretome were evaluated.

***Results:*** Our results indicate that MCF-7 cells express significant amount of IL-6 and IL-8. Moreover, significant amounts of IL-1a, IL-1b, IL-8, IL-6 and GM-CSF were detected in secretome of hWJ-MSCs. Furthermore, IL-1a, IL-2 and IL-4 were expressed significantly by MCF-7 cells after their treatment with hWJ-MSCs-derived secretome.

***Conclusion:*** According to our findings, the hWJ-MSCs derived secretome contains different cytokines which can exert either anti-tumor or tumorigenic effects.

## Introduction

Cancer is a leading cause of mortality in human societies. Different therapies such as chemotherapy, radiotherapy, hormone therapy and antibody based therapies are used for many types of cancers in the clinic.^1^ None of these therapies has been able to fully cure cancer, and each of them has a partial therapeutic effect.^2^ Hence, many studies are underway to find other therapeutic approaches and, more importantly, effective therapeutic strategies. Recently, many studies have been suggested that mesenchymal stem cells (MSCs) could be used as a therapeutic approach to treating cancers and many other diseases.^3,4^ Today, different types of MSCs have been characterized, including bone marrow MSCs (BM-MSCs), adipose tissue MSCs, umbilical cord-derived stem cells, peripheral blood-derived MSCs and many others.^4,5^ The use of MSCs, especially in regenerative medicine, has made remarkable progress and the positive effect of these cells on the repair of damaged tissues has been reported.^6,7^ Many studies have shown that stem cells have a high tendency (tropism) toward inflammatory or tumor environment in unknown mechanisms.^8,9^ However, contradictory results have been achieved using the stem cells for the treatment of various types of cancers. Many studies have shown that these cells can have both pro-tumorigenic and anti-tumor properties.^10-13^ Therefore, the use of these cells in the treatment of cancers acts like a double sword.^14^ Among various sources of these cells, it has been shown that umbilical-cord MSCs (hWJ-MSCs) can inhibit tumor cells growth.^6,15^ One of the important features of these cells that distinguishes them from other types is the high capacity of such cells to replicate.^16^ Also, hWJ-MSCs can be easily cultured and reproduced in large scales and could be used for treatment from allogeneic donors.^6^ One of the most important characteristics of these cells, which cause their allogeneic application, is their low immunogenicity. These cells express MHC-I molecules, while they do not express MHC-II molecules.^16^ Also these cells have high tropism toward inflammatory environments like tumor microenvironment.^17^ Due to concerns about the potential pro-tumorigenic effects of these cells, various studies have shown that secretome and exosomes derived from these cells could be useful in the treatment of different cancers instead of cell based therapies.^17-19^ Also, compared to stem cell therapy, cell free therapeutic approach is much more economical and practical for use in the clinic.^20^ In this regard using secretome of the stem cells as therapeutic agents has faced many challenges. Studies have shown that like stem cell based therapies the effects of secretome can be both pro-inflammatory and anti-inflammatory. For example, the use of conditioned media obtained from bone marrow derived stem cells showed that these substances have antitumor effects on non-small cell lung cancer cells, while the same agents have supportive effects against bone marrow cancer or multiple myeloma.^21,22^ Another study conducted using secretome of stem cells derived from adipose tissue showed that these materials had no effect on glioblastoma cancer cells.^23^ Also, the antitumor effects of the secretome of the human uterine tissue-derived stem cells conditioned media (hUCESC-CM) have been shown.^17^

These results indicate that the anti-tumor or pro-tumorigenic effects of the MSCs derived secretome are both depended on the stem cells types which secretome obtained from, as well as the type of tumor.^17^ Cytokines are one of the major components of cell derived secretome, which may play an important role in inducing these effects.^24^ It has been shown that the secretome of ADSC-CM contains tumor supportive factors, including FGF-4, IL-6, IL-6R, CCL7 and VEGF-D, while hUCESC-CM secretome mainly contain antitumor agents such as the TNF cytokine, FLT3-Ligand and CXCL10.^17^ In this study we want to examine the presence of different cytokines in secretome of hWJ-MSCs and their effects on cytokine expression profile of the MCF-7 breast cancer cells both before and after treatment.

## Materials and Methods

### Isolation, culture and expansion of mesenchymal stem cells

MSCs were isolated from the human umbilical cord. For this purpose, umbilical cord tissues with a length of approximately 20 cm were collected from donors who referred to Isfahan Family Specialty Clinics and undergone cesarean section. All the participants voluntarily involved in this study by giving a consent letter based on the ethical guidelines of Isfahan University of Medical Sciences. The tissues were collected and immediately sterilized in a sterile saline containing 50 μg/mL streptomycin and 50 IU/mL penicillin and 0.125 μg/mL amphotericin B (PAA Laboratories GmbH, Austria) and then transferred to laboratory. Under sterile condition of laminar flow hood, the umbilical cord was cut into 1.5 cm pieces. After separating the blood vessels, the gelatinous tissue of Wharton Jelly surrounding the vessels was excised and plated on 6 cm sterile plates containing DMEM medium (low glucose medium enriched with 10% FBS and 1% pen/strep; Gibco, Eggenstein, Germany). Plates were then incubated in a 37°C humidified incubator containing 5% CO2. The medium was changed every three days to remove dead cells and debris from tissues. During this time, the cells migrated out of the tissues of the umbilical cord and proliferated in the plate to form a confluent monolayer. The cells were then sub-cultured and expanded for further experiments.

### Characterization of mesenchymal stem cells

The hWJ-MSC examined by optical microscope and morphologically looked as fibroblastic adherent cells. Also the cells were further evaluated for the presence of CD14, CD34, CD45, CD73, CD90, and CD105 markers using flow cytometry. Furthermore, the differentiation capability of these MSCs to different lineages has been previously reported by our group elsewhere which have used the same source of cells.^25,26^ For the experiments, cells were washed twice with PBS to remove cell debris and then cells were detached using trypsin 0.25%. Cells were stained for 25 min at 4°C using fluorescence-labeled or PE labeled anti human antibodies (Biolegend, San Diego, CA, USA). Moreover, mouse IgG antibodies conjugated with identical concentrations of FITC and PE were used as negative control. Each flow cytometry experiment was performed with at least 10 000 events using FACS Caliber^®^ flowcytometer (BD biosciences, USA).

### Determination ofsecretomeconcentration

To obtain optimal concentration of secretome derived from hWJ-MSCs cells of the 4thpassage with 80%-90% confluency in the 75-cm flask washed twice with phosphate buffer solution (PBS). Then, 10 mL of serum free DMEM medium was added to each flask and incubated for 48 hours. After incubation, the supernatant was collected and centrifuged for 5 minutes to remove cell debris and dead cells. Finally, the supernatant was dried using a freeze-dryer device and stored at -80°Cuntil use. In order to find the optimum concentration of secretome derived from hWJ-MSCs, we examined the IC50 of these secretome in logarithmic concentrations (100, 10, 1, 0.1and 0.01 mg/mL; data are not shown) using MTT assay. In this regard the cancer cells were incubated for 24 hours and then 20 microliters of each concentration was added to each well containing 180 μL of the DEMEM and the plate was incubated for further 48 hours. Finally, the plates were analyzed at 570 nm by ELISA microplate reader.

### Cytokine array

The cytokine array method was used to determine the presence of different cytokines in secretome derived from hWJ-MSCs and their effect on cytokine expression profile of MCF-7 tumor cells. To this end, a cytokine array kit (Multi-Analyte ELIS Array Kit) coated with 12 specific antibodies against 12 different cytokines including IL-1A, IL-1B, IL-2, IL-4, IL-6, IL-8, IL-10, IL-12, IL-17A, IFNY, TNFα and GM-CSF was used (Qiagen, Hilden, Germany). Briefly, the supernatant of 6 different treatment groups including MCF-7 tumor cells treated with 20 mg/mL of secretome (MCF-7 + MSC20), treated with 10 mg/mL of secretome (MCF-7 + MSC10), treated with 0.1 mg/mL of secretome (MCF-7 + MSC0.1), 20 mg/mL of secretome and 10 mg/mL of MSCs secretome were collected. All the experiments were performed alongside the positive and negative controls according to manufacturer’s instruction. The negative control was 50 μL of the dilution buffer and the positive control was 50 μL of standard antigen cocktail containing the subjected 12 cytokines provided by kit. Then, 50 μL of the samples and controls were added to different wells and incubated for 2 hours in 37°C. After washing three times with PSBT20, 100 μL of detection antibody solution was added and incubated for further 1 hour. It was then washed again with PBST20 (3 times) and 100 μL of avidin-HRP were added and incubated for 30 min. Afterwards, 100 μL of development solution was added and incubated for 15 min in a dark condition, and finally 100 μL of stop solution was added, and its absorbance was measured at 450 nm. In the next step the concentration each cytokine (which was identified in the abovementioned samples) was measures using another ELISA kit according to the manufacturer’s instructions (eBioscience, Affymetrix, CA, USA). The generated signal was proportional to the protein content of sample.

### Data analysis

All experiments were performed independently in triplicate. The data were analyzed with one-way ANOVA test using SPSS software (version 20, IBM SPSS statistics data editor). Differences between groups were significant at *P* <0.05. The results are presented as mean ± SD.

## Results and Discussion

### Proliferation ofhWJ-MSCs

MSCs were isolated, cultured and expanded successfully according to the procedure mentioned in methods and materials. It worth to mention that MSCs from umbilical cord of mothers who had naturally given birth were removed faster than cesarean section (unpublished data).

### Flowcytometric analysis ofhWJ-MSCs

**Figure 1:**
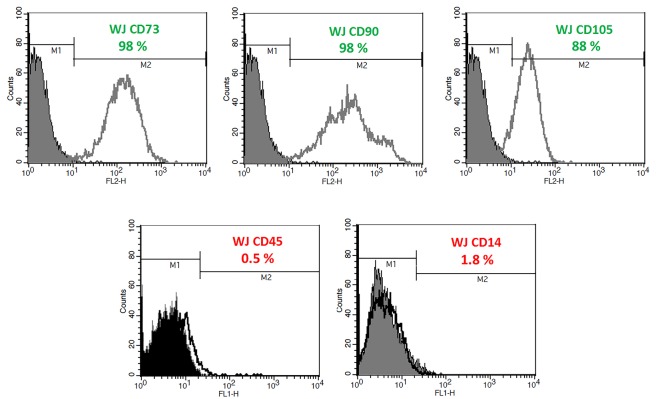
Characterization of stem cells isolated from the human umbilical cord using flow cytometry. Cells were analyzed using FITC and PE labeled anti-CD105, -CD90, and anti-CD73 antibodies. Also anti-CD14 and -CD45 antibodies were used to discriminate MSCs from cells with hematopoietic origin.

The hWJ-MSCs were examined by optical microscopy and found to be looked as fibroblastic adherent cells. Also the cells were further evaluated for the presence of CD14, CD34, CD45, CD73, CD90, and CD105 markers using flow cytometry and were found to be positive for CD105, CD90, CD73 and CD34 surface markers. Moreover, these cells were negative in terms of CD14 and CD45 markers, which indicate the non-hematopoietic origin of these cells. Our flow cytometric results (Figure 1) clearly confirm that the cells isolated from the umbilical cord are more than 95% positive in terms of CD105, CD90 and CD73 markers, and also do not express CD14 and CD45 markers. Also, the differentiation capability of hWJ-MSCs cells to different lineages has been verified previously by our team which used same source of cells.^25^

### Effect ofhWJ-MSCs derivedsecretomeon MCF-7 cells

According to MTT assay (data will be published elsewhere) the concentration of hWJ-MSCs secretome which can inhibit 50% of MCF-7 cells (IC50) was found to be 10 mg/mL. This concentration along with a higher concentration of secretome (20 mg/mL) was chosen for further experiments.

### Cytokine expression profile ofhWJ-MSCs and MCF-7 cells

**Figure 2:**
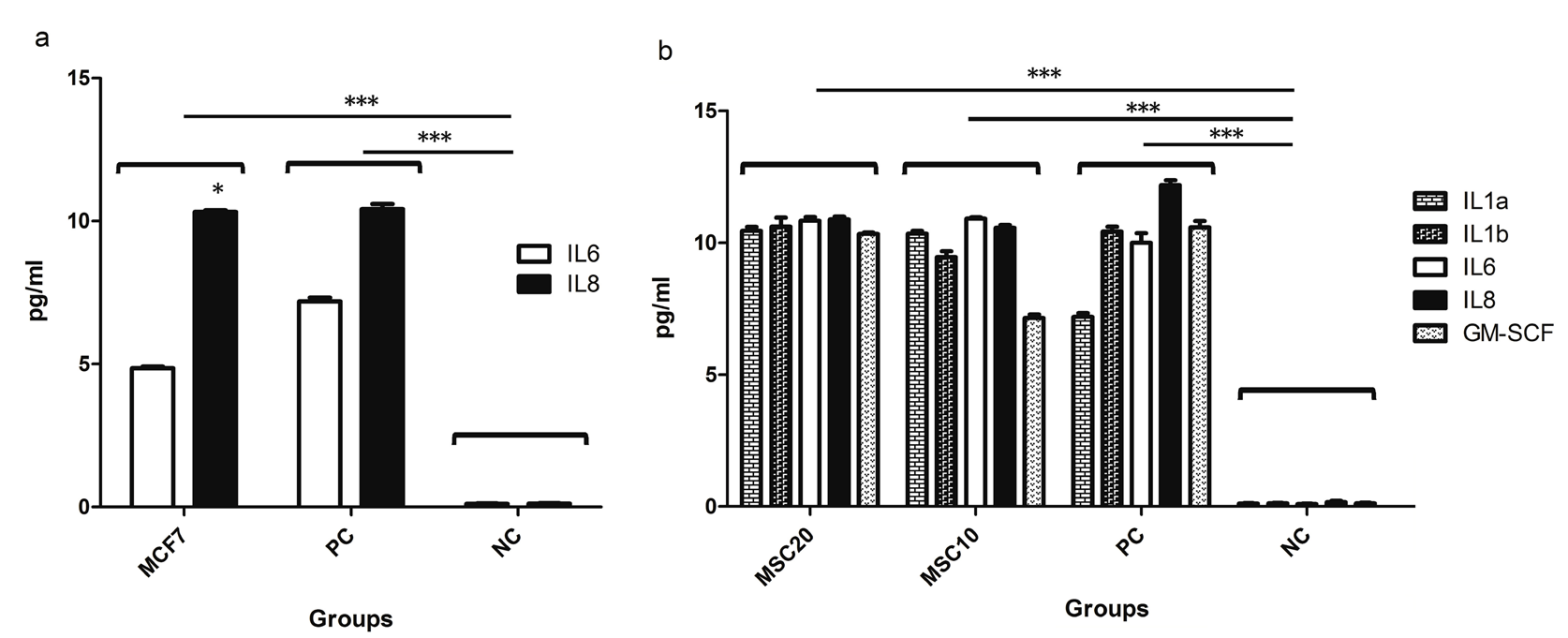
Cytokine expression profile of MCF-7tumor cells and cytokine expression profile of hWJ-MSCs assessed using different concentrations of freeze-dried secretome of MSC cells (MSC20: 20 mg/mL and MSC10: 10 mg/mL). The expression amount of other cytokines was negligible. PC means Positive Control; NC stands for Negative Control. * stands for p value <0.05 and *** stands for p value <0.001. For PC and NC see cytokine array section in method and materials.

The supernatant of MCF-7 tumor cells as well as secretome of hWJ-MSCs were examined to evaluate expression of 12 different cytokines consequently. According to our results, the MCF-7 cells express only two cytokines of IL-6 and IL-8 (almost in double amount) significantly in their supernatant compared to negative control (Figure 2a), while IL1a, IL1b, IL6, IL8, and GM-CSF were expressed in hWJ-MSCs secretome in significant amounts (Figure 2b).

### Effect ofhWJ-MSCssecretomeon cytokine expression profile of MCF-7 tumor cells

**Figure 3:**
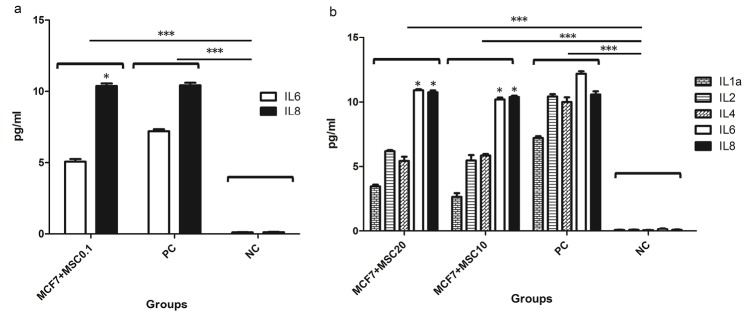
Cytokine expression profile of MCF-7 cells treated with 0.1 mg/mL of freeze dried secretome of hWJ-MSCs (MCF-7+MSC0.1). Cytokines expression profile of MCF-7 cells after treatment with 20 mg/mL (MCF-7 + MSC20) and 10mg/mL (MCF-7 + MSC10) of freeze-dried secretome of hWJ-MSCs. * stands for p value <0.05 and *** stands for p value < 0.001.

MCF-7 tumor cells were treated with freeze-dried of hWJ-MSCs secretome at IC50 concentration (10 mg/mL indicated as MSC10) along with a lower and a higher concentration (0.1 mg/mL indicated as MSC0.1 and 20mg/mL indicated as MSC20, respectively). Then MCF-7 cells supernatants were examined to detect 12 different cytokines available in our kits. As shown in Figure 3. The cytokine expression pattern of MCF-7 cells were treated with lower concentration of MSC secretome (0.1 mg/mL, Figure 3a) was exactly similar to untreated MCF-7 cells (Figure 2a) where expressed only IL-6 and IL-8; where as in higher concentration (10 and 20 mg/mL) this pattern were changed and other interleukins including IL-1a, IL-2 and IL-4 were also expressed by MCF-7 (Figure 3b) which were not expressed by untreated MCF-7 cells.

It has been demonstrated that stem cells have potential therapeutic effects in treatment of various diseases, including early stages of cancers and their metastatic forms.^27^ In this regard, several studies have shown that hWJ-MSCs can significantly reduce the growth of breast cancer, lung cancer, and murine cell line of the pancreatic carcinoma.^17,28,29^ However, there are other evidences which show that stem cells can also lead to increased growth of tumor cells.^30^ These results raise concerns about the use of stem cells for cancer treatment. Nowadays many studies try to use secretome and exosomes derived from these cells to treat various diseases, including cancers, instead of using cell based therapies.^31,32^ In fact, stem cells, with two main mechanisms induce their anti-tumor properties; by direct contact with tumor cells, and by secretion of various factors (cytokines and etc.).^29^ In the present study we aimed to examine the different cytokines present in the secretome of hWJ-MSCs and to evaluate the cytokine expression pattern of MCF-7 tumor cells before and after treatment with different concentrations of hWJ-MSCs derived secretome. As illustrated in Figure 2a, the MCF-7tumor cells express IL-6 and IL-8 cytokines before any treatment. These results are consistent with studies have been shown that increased IL-6 expression by tumor cells is associated with malignancy and poor patient condition.^33^ Also, it has been reported that IL-6 expressed by cancer cells increases their proliferation, differentiation, and resistance to apoptosis in many types of tumors.^34^ Moreover, high expression of IL-8 by tumor cells is associated with angiogenesis and drug resistance of tumors.^34,35^ As shown in Figure 2b, our results showed that IL-6,IL-8, along with high levels of IL-1a, IL-1b and GM-CSF cytokines are secreted from hWJ-MSCS cells and are presented in their secretome. Regarding to our results and the key role of IL-6 and IL-8 in the proliferation of tumor cells, it seems that one of the mechanisms by which stem cells contribute to tumor progression is secretion of these two cytokines. Other studies also show that the presence of IL-1 in the tumor microenvironment can lead to tumor malignancy and worsen the situation.^34^ It has been proven that IL-1 induces mutagenesis in cancer cells and thus results in heterogeneity in the population of tumor cells and also improves their resistance to treatments.^34^ According to our results, it can be expected that IL-1expressed in secretome of the hWJ-MSCs may lead to tumor progression. Interestingly, according to Figures 3a and 3b, our results indicate that the treatment of the MCF-7 tumor cells with secretome of the hWJ-MSCS stem cells could alter the cytokine expression pattern of the tumor cells. As shown in Figure 3b, in addition to the IL-6 and IL-8 treated, MCF-7 cells also produce significant amounts of IL-1a, IL-2, and IL-4. IL-1a is most likely secreted autocrinely and could improve tumor progression by inhibition of the immune system. However, in the case of IL-4, many studies have shown that the secretion of this cytokine in tumor microenvironment induces the M2 macrophages, which increase tumor progression by secretion of proteases such as catepsin.^34^ According to Figure 3b, treated MCF-7 cells expressed significant amount of IL-2, which has been proven that it has anti-tumor properties and lead to increased antitumor activity of the immune cells.^33,34^ Indeed, IL-2 is a key cytokine in increasing and differentiation of anti-tumor T and NK cells. For this reason, there are many studies today that emphasize on the importance of using IL-2 as a treatment for patients with cancer.^35^ Extensive clinical trials have been used IL-2 in the treatment of metastatic melanoma, renal carcinoma, and intestinal cancer, which associated with promising results. Our results show that secretion of IL-2 from MCF-7 cells in response to secretome of Wharton’s jelly stem cell as well as the secretion of GM-CSF from stem cells themselves could lead to tumor regression.GM-CSF is essential for the differentiation of some cells and also responsible for processing and presenting tumor antigens for the priming of antitumor cytotoxic T lymphocytes.^36^ It may be concluded that secretome of stem cells may alter the cytokine expression pattern of tumor cells which subsequently could lead to tumor progression or regression. It has to be mentioned that the GM-CSF was detected in supernatant of hWJ-MSCs, while this cytokine was not found in supernatant of MCF-7 tumor cells (Figure 3), which may emphasize that the tumor cells did not secrete GM-CSF.

## Conclusion

Our results showed that both anti-tumor and tumor supportive cytokines present in secretome of the hWJ-MSCS. Moreover, according to our findings, cytokine expression pattern of tumor cells may alter after their treatment with secretome of stem cells. Thus, we speculate that the subsequent dominancy in two types of these cytokines in tumor microenvironment could lead to tumor progression or regression. Finally, our results strongly support this idea that soluble factors such as cytokines present in secretome of stem cells could result in different anti-tumor or tumorigenic responses.

## Ethical Issues

Not applicable.

## Conflict of Interest

The authors declare that they have no conflict of interest.

## Acknowledgments

This study was supported by Iran National Science Foundation (INSF; grant No. 91004356).

## References

[1] Gotwals P, Cameron S, Cipolletta D, Cremasco V, Crystal A, Hewes B (2017). Prospects for combining targeted and conventional cancer therapy with immunotherapy. Nat Rev Cancer.

[2] Dagogo-Jack I, Shaw AT (2018). Tumour heterogeneity and resistance to cancer therapies. Nat Rev Clin Oncol.

[3] Sun XY, Nong J, Qin K, Warnock GL, Dai LJ (2011). Mesenchymal stem cell-mediated cancer therapy: A dual-targeted strategy of personalized medicine. World J Stem Cells.

[4] Li X, Bai J, Ji X, Li R, Xuan Y, Wang Y (2014). Comprehensive characterization of four different populations of human mesenchymal stem cells as regards their immune properties, proliferation and differentiation. Int J Mol Med.

[5] Wang LT, Ting CH, Yen ML, Liu KJ, Sytwu HK, Wu KK (2016). Human mesenchymal stem cells (MSCs) for treatment towards immune- and inflammation-mediated diseases: review of current clinical trials. J Biomed Sci.

[6] Batsali AK, Kastrinaki MC, Papadaki HA, Pontikoglou C (2013). Mesenchymal stem cells derived from Wharton’s Jelly of the umbilical cord: biological properties and emerging clinical applications. Curr Stem Cell Res Ther.

[7] McGuirk JP, Smith JR, Divine CL, Zuniga M, Weiss ML (2015). Wharton’s Jelly-Derived Mesenchymal Stromal Cells as a Promising Cellular Therapeutic Strategy for the Management of Graft-versus-Host Disease. Pharmaceuticals (Basel).

[8] Wang Y, Chen X, Cao W, Shi Y (2014). Plasticity of mesenchymal stem cells in immunomodulation: pathological and therapeutic implications. Nat Immunol.

[9] Chulpanova DS, Kitaeva KV, Tazetdinova LG, James V, Rizvanov AA, Solovyeva VV (2018). Application of Mesenchymal Stem Cells for Therapeutic Agent Delivery in Anti-tumor Treatment. Front Pharmacol.

[10] Zhang CL, Huang T, Wu BL, He WX, Liu D (2017). Stem cells in cancer therapy: opportunities and challenges. Oncotarget.

[11] Gomes JPA, Assoni AF, Pelatti M, Coatti G, Okamoto OK, Zatz M (2017). Deepening a Simple Question: Can MSCs Be Used to Treat Cancer?. Anticancer Res.

[12] Melzer C, Yang Y, Hass R (2016). Interaction of MSC with tumor cells. Cell Commun Signal.

[13] Klopp AH, Gupta A, Spaeth E, Andreeff M, Marini F 3rd (2011). Concise review: Dissecting a discrepancy in the literature: do mesenchymal stem cells support or suppress tumor growth?. Stem Cells.

[14] Lee HY, Hong IS (2017). Double-edged sword of mesenchymal stem cells: Cancer-promoting versus therapeutic potential. Cancer Sci.

[15] Ramdasi S, Sarang S, Viswanathan C (2015). Potential of Mesenchymal Stem Cell based application in Cancer. Int J Hematol Oncol Stem Cell Res.

[16] Liu L, Chai J, Han Y, Sun T, Li D, Zhao J (2011). [Research progress of biological characteristics and advantages of Wharton’s jelly-mesenchymal stem cells]. Zhongguo Xiu Fu Chong Jian Wai Ke Za Zhi.

[17] Vizoso FJ, Eiro N, Cid S, Schneider J, Perez-Fernandez R (2017). Mesenchymal Stem Cell Secretome: Toward Cell-Free Therapeutic Strategies in Regenerative Medicine. Int J Mol Sci.

[18] Madrigal M, Rao KS, Riordan NH (2014). A review of therapeutic effects of mesenchymal stem cell secretions and induction of secretory modification by different culture methods. J Transl Med.

[19] Purnamawati Purnamawati, Pawitan JA, Rachman A, Liem IK, Wanandi SI (2017). Secretomes of adipose and umbilical cord-derived stem cells affect ALDH1A1 expression in breast cancer stem cells. Adv Sci Lett.

[20] Osugi M, Katagiri W, Yoshimi R, Inukai T, Hibi H, Ueda M (2012). Conditioned media from mesenchymal stem cells enhanced bone regeneration in rat calvarial bone defects. Tissue Eng Part A.

[21] Attar-Schneider O, Zismanov V, Drucker L, Gottfried M (2016). Secretome of human bone marrow mesenchymal stem cells: an emerging player in lung cancer progression and mechanisms of translation initiation. Tumour Biol.

[22] Marcus H, Attar-Schneider O, Dabbah M, Zismanov V, Tartakover-Matalon S, Lishner M (2016). Mesenchymal stem cells secretomes’ affect multiple myeloma translation initiation. Cell Signal.

[23] Onzi GR, Ledur PF, Hainzenreder LD, Bertoni AP, Silva AO, Lenz G (2016). Analysis of the safety of mesenchymal stromal cells secretome for glioblastoma treatment. Cytotherapy.

[24] Kontostathi G, Zoidakis J, Makridakis M, Lygirou V, Mermelekas G, Papadopoulos T (2017). Cervical cancer cell line secretome highlights the roles of transforming growth factor-beta-induced protein ig-h3, peroxiredoxin-2, and NRF2 on cervical carcinogenesis. Biomed Res Int.

[25] Hendijani F, Haghjooy Javanmard SH, Rafiee L, Sadeghi-Aliabadi H (2015). Effect of human Wharton’s jelly mesenchymal stem cell secretome on proliferation, apoptosis and drug resistance of lung cancer cells. Res Pharm Sci.

[26] Hendijani F, Haghjooy Javanmard SH, Sadeghi-Aliabadi H (2015). Human Wharton’s jelly mesenchymal stem cell secretome display antiproliferative effect on leukemia cell line and produce additive cytotoxic effect in combination with doxorubicin. Tissue Cell.

[27] Parekkadan B, Milwid JM (2010). Mesenchymal stem cells as therapeutics. Annu Rev Biomed Eng.

[28] Donders R, Bogie JFJ, Ravanidis S, Gervois P, Vanheusden M, Maree R (2018). Human Wharton’s jelly-derived stem cells display a distinct immunomodulatory and proregenerative transcriptional signature compared to bone marrow-derived stem cells. Stem Cells Dev.

[29] Tamura M, Kawabata A, Ohta N, Uppalapati L, Becker KG, Troyer D (2011). Wharton’s jelly stem cells as agents for cancer therapy. Open Tissue Eng Regen Med J.

[30] Shinagawa K, Kitadai Y, Tanaka M, Sumida T, Kodama M, Higashi Y (2010). Mesenchymal stem cells enhance growth and metastasis of colon cancer. Int J Cancer.

[31] Makridakis M, Roubelakis MG, Vlahou A (2013). Stem cells: insights into the secretome. Biochim Biophys Acta.

[32] Wang J, Zheng Y, Zhao M (2016). Exosome-Based Cancer Therapy: Implication for Targeting Cancer Stem Cells. Front Pharmacol.

[33] Yoshimoto T, Morishima N, Okumura M, Chiba Y, Xu M, Mizuguchi J (2009). Interleukins and cancer immunotherapy. Immunotherapy.

[34] Setrerrahmane S, Xu H (2017). Tumor-related interleukins: old validated targets for new anti-cancer drug development. Mol Cancer.

[35] Brat DJ, Bellail AC, Van Meir EG (2005). The role of interleukin-8 and its receptors in gliomagenesis and tumoral angiogenesis. Neuro Oncol.

[36] Yan WL, Shen KY, Tien CY, Chen YA, Liu SJ (2017). Recent progress in GM-CSF-based cancer immunotherapy. Immunotherapy.

